# Resonance Raman enhancement by the intralayer and interlayer electron–phonon processes in twisted bilayer graphene

**DOI:** 10.1038/s41598-021-96515-0

**Published:** 2021-08-26

**Authors:** M. V. O. Moutinho, G. S. N. Eliel, A. Righi, R. N. Gontijo, M. Paillet, T. Michel, Po-Wen Chiu, P. Venezuela, M. A. Pimenta

**Affiliations:** 1 Núcleo Multidisciplinar de Pesquisas em Computação-NUMPEX-COMP, Campus Duque de Caxias, Universidade Federal do Rio de Janeiro, Duque de Caxias, RJ Brazil; 2 Instituto de Física, Universidade Federal da Bahia, Salvador, BA Brazil; 3 Departamento de Física, Universidade Federal de Minas Gerais, Belo Horizonte, MG Brazil; 4 Laboratoire Charles Coulomb, CNRS, University of Montpellier, 34095 Montpellier, France; 5 National Tsing Hua University, Hsinchu, 30013 Taiwan; 6 Instituto de Física, Universidade Federal Fluminense, Niterói, Rio de Janeiro Brazil

**Keywords:** Optical properties and devices, Electronic properties and materials

## Abstract

Twisted bilayer graphene is a fascinating system due to the possibility of tuning the electronic and optical properties by controlling the twisting angle $\theta$ between the layers. The coupling between the Dirac cones of the two graphene layers gives rise to van Hove singularities (vHs) in the density of electronic states, whose energies vary with $\theta$. Raman spectroscopy is a fundamental tool to study twisted bilayer graphene (TBG) systems since the Raman response is hugely enhanced when the photons are in resonance with transition between vHs and new peaks appear in the Raman spectra due to phonons within the interior of the Brillouin zone of graphene that are activated by the Moiré superlattice. It was recently shown that these new peaks can be activated by the intralayer and the interlayer electron–phonon processes. In this work we study how each one of these processes enhances the intensities of the peaks coming from the acoustic and optical phonon branches of graphene. Resonance Raman measurements, performed in many different TBG samples with $\theta$ between $4^{\circ }$ and $16^{\circ }$ and using several different laser excitation energies in the near-infrared (NIR) and visible ranges (1.39–2.71 eV), reveal the distinct enhancement of the different phonons of graphene by the intralayer and interlayer processes. Experimental results are nicely explained by theoretical calculations of the double-resonance Raman intensity in graphene by imposing the momentum conservation rules for the intralayer and the interlayer electron–phonon resonant conditions in TBGs. Our results show that the resonant enhancement of the Raman response in all cases is affected by the quantum interference effect and the symmetry requirements of the double resonance Raman process in graphene.

## Introduction

The superposition of adjacent layers in twisted bilayer graphene (TBG) generates a Moiré pattern superlattice whose size depends on the twisting angle $\theta$ between the layers. In momentum space, the interaction between the Dirac cones of each layer gives rise to van Hove singularities (vHs) in the density of electronic states, whose energies also depends on $\theta$. The possibility of controlling the twisting angle $\theta$ in TBG opens thus fascinating possibilities of developing tunable opto-electronic devices^1–4^. The interest in the study of TBGs was recently revived after the observation of superconductivity in samples with $\theta$ close to 1.1$^{\circ }$^5,6^. In this so-called magic angle, the interaction between the cones generates flat electronic bands with energy width of few meV and, in this regime, the electron–electron interactions can dominate the many body interactions and give rise to correlated phenomena such as the superconductivity.

Raman spectroscopy is a powerful and extremely useful technique to characterize any kind of graphene related system^7–16^. For monolayer graphene, only one optical phonon mode is active in the first-order Raman spectrum and it gives rise to the G band around 1580 ${\hbox {cm}}^{-1}$. In the case of TBGs, two new phenomena are observed through the Raman spectra: (i) a huge enhancement of the G band when the photons of the Raman process are in resonance with the vHs in the valence and conduction bands^17–19^ and (ii) the appearance of extra peaks in the spectra associated with phonons in the interior of Brillouin zone (BZ) of monolayer graphene that are activated by the Moiré pattern in the spectra of TBGs^20–26^. Other Raman bands are also observed in graphene systems due to the double-resonance (DR) mechanism, such as the 2D band and the disorder-induced D band, being the dependence of the band positions and intensities on the laser excitation energy their main characteristics^17,27^.

Since the first report by Gupta et al. in 2010^20^ several works in the literature have also reported the appearance of new peaks in the Raman spectra of TBGs, and have shown that their frequencies depend on the twisting angle $\theta$^21–26^. These peaks come from phonons within the interior of the Brillouin zone (BZ) of monolayer graphene that have the wavevector ${\mathbf{q}}_{{\mathbf{M}}}$ of the Moiré superlattice reciprocal space. They are folded to the centre of the reduced BZ of the Moiré superlattice and become Raman active. It was shown recently that there are two different mechanisms that enhance the Moiré peaks in the TBG Raman spectra^28^. In one case, the enhancement of these peaks occurs in the same range of excitation energies where the G band is hugely enhanced, and, in the other case, the enhancement occurs for excitation laser energies where the G band is not enhanced. These results were explained considering the existence of two resonance mechanisms: the intralayer and interlayer electron–phonon (el–ph) interaction processes. The intralayer process involves electronic scattering between states in the Dirac cone of the same layer and in the interlayer case the scattering occurs between states from different layers^28^.

In the present work, we study experimentally and theoretically the enhancement of the Moiré peaks of TBGs coming from the acoustic and optical phonon branches of graphene by both the intralayer and the interlayer el–ph processes. We present experimental results obtained from many different TBG samples with small and intermediate twisting angles (between $4.3^{\circ }$ and $16.3^{\circ }$) and using several different laser excitation energies in the visible and NIR ranges (between 1.39 eV and 2.71 eV). Our results show that the enhancement of Moiré peaks from the different acoustic and optical branches of graphene by the intralayer and the interlayer electron–phonon processes are distinct. The experimental results are explained by calculations based on double-resonance Raman processes in graphene^7,27^ by imposing the momentum conservation for the intralayer and interlayer el–ph processes. We also show that the positions of the Moiré peaks from the acoustic phonon branches can be used to measure accurately the twisting angle $\theta$ of a TBG sample.

## Results and discussion

### Acoustic and optical phonons of graphene in TBG Raman spectra

**Figure 1:**
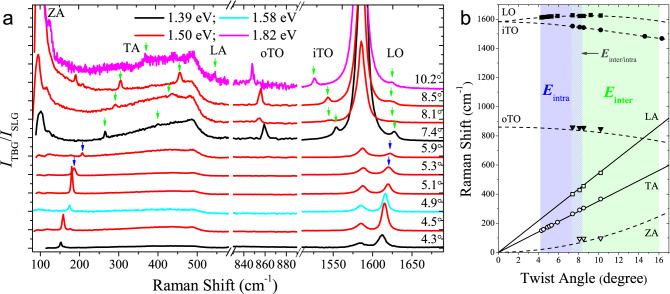
(**a**) Raman spectra of ten TBG samples with different angles between 4.3$^\circ$ and 10.2$^\circ$. The spectra were acquired using four different laser lines, represented by different colours in the figure, but only those presenting the strongest resonance effect are displayed in the figure. The spectra is shown in three regions: (i) the acoustic modes (ZA, TA and LA), (ii) the out-of-plane TO phonon (oTO) and (iii) the optical modes close to the G band (iTO and LO). All spectra with $\theta \ge 7.4^{\circ }$ were multiplied by $10\,\times$ in the acoustic and the oTO regions for a better visualisation. (**b**) Positions of all peaks extracted from all Raman spectra plotted as a function of $\theta$. The twisting angles were determined by the LA and TA phonon branches according to dispersion curves (full black lines) obtained by Cong et al.^29^ (see text). The dashed black lines are theoretical curves of the TBG phonon frequencies^27^ as a function of the twisting angle^28^. The blue (green) stripe highlights the range of angles of the TBGs investigated in this work that are activated by the intralayer (interlayer) el–ph process.

Figure 1a shows the Raman spectra of 10 different samples of TBG with twisting angles between 4.3$^\circ$ and 10.2$^\circ$, which have been measured with different laser excitation energies in the NIR range: 1.39 eV (890 nm), 1.50 eV (827 nm), 1.58 eV (785 nm), 1.70 eV (728 nm) and 1.82 eV (680 nm). The spectra depicted in Fig. 1a correspond to the excitation energy that gives rise to the strongest resonance Raman effect for each sample. We can observe that the spectra can be separated in three spectral ranges: (i) between 100 ${\hbox {cm}}^{-1}$ and 600 ${\hbox {cm}}^{-1}$ we observe the peaks associated with the acoustic phonon branches (ZA, TA and LA), (ii) around 850 ${\hbox {cm}}^{-1}$ we observe the peaks associated with the out-of-plane transverse optical (oTO) phonon mode and (iii) in the range 1400-1700 ${\hbox {cm}}^{-1}$ we observe the G band (1580 ${\hbox {cm}}^{-1}$) and the peaks originated from the in-plane transverse optical (iTO) phonon branch and the longitudinal optical (LO) phonon branch, respectively below and above the G band.

The twisting angles $\theta$ were initially estimated from the optical images of the TBG samples, where we can observe the crystalline edges of the two graphene layers, as previously reported by Ribeiro et al.^19^. However, the angle determined using this method is not accurate and the uncertainty is of the order of 1$^{\circ }$. The accuracy in the angles determination was improved by considering the dependence of acoustic phonon frequencies (TA and LA) on the twisting angle $\theta$, since the dispersive behavior of these branches provides the determination of $\theta$ with an accuracy of the order of 0.1$^{\circ }$.

Cong et al.^29^ have probed the LA and TA phonon branches of monolayer graphene near the $\Gamma$ point by double resonant Raman scattering of combination phonon modes and the overtone 2${\hbox {D}}^{\prime }$ mode. They obtained the values of 12.9 km ${\hbox {s}}^{-1}$ and 19.9 km ${\hbox {s}}^{-1}$ for the sound velocities of TA and LA branches, respectively. For TBG, the twisting angle may be obtained by the expression $\sin (\theta /2)=f\sqrt{3}a/4v$, being *a* the lattice parameter of monolayer graphene, *f* the frequency and *v* the sound velocity of the TA or LA branch. The twisting angle $\theta$ is then obtained from the experimental frequencies of the TA and LA phonons using the solid lines in Fig. 1b, where the TA peak position is related to the twisting angle by the expression $f_{{\text{TA}}}$ = 4040 $\sin (\theta /2)$ ${\hbox {cm}}^{-1}$ while the LA curve is given by $f_{{\text{LA}}}$ = 6232 $\sin (\theta /2)$ ${\hbox {cm}}^{-1}$. In the case of a TBG sample with the magic angle of 1.1$^{\circ }$, the TA and LA peaks are expected to appear in the Raman spectrum around 39 ${\hbox {cm}}^{-1}$ and 60 ${\hbox {cm}}^{-1}$, respectively. However, as we will show below, the enhancement of these peaks is only expected to be observed for excitation energies in the mid-IR range, below the existent laser energies in the NIR range.

Figure 1b shows the positions of all Raman peaks of the investigated TBG samples as a function of the twisting angle $\theta$. The dispersion relations for the ZA, oTO, iTO and LO phonons are also plotted in Fig. 1b as dashed curves and were calculated by means of density functional theory as discussed in Ref.^27^. The precise determination of the ZA peak positions is affected by the fact that the spectrometer cuts the scattered light below 100 ${\hbox {cm}}^{-1}$. Therefore, we only plotted the data of the highest angle samples, where the peak position is above 100 ${\hbox {cm}}^{-1}$.

### Resonance Raman enhancement by the intralayer and interlayer el–ph processes

**Figure 2:**
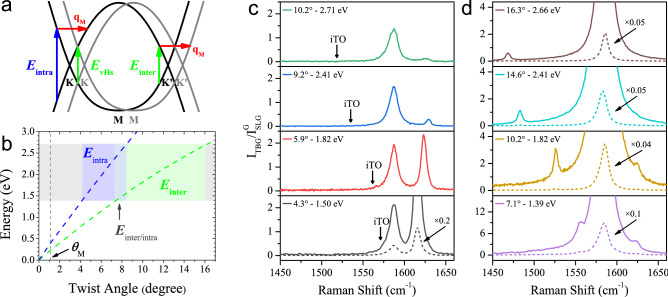
(**a**) Schematic diagram of the intralayer (blue arrow) and interlayer (green arrow) processes in the Dirac cones of two graphene layers. The grey (black) curves represent the electronic bands and the Dirac cones of layer A (layer B), and the red arrow represents the phonon wave vector, which corresponds to the unit vector ${\mathbf{q}}_{{\text {M}}}$ of the Moiré reciprocal lattice. (**b**) Calculated dependence of the intralayer (dashed blue) and interlayer (dashed green) resonance energies, respectively, on the twisting angle^28^. The horizontal gray stripe denotes the range of excitation energies used in this work (1.39–2.71 eV) and the blue (green) rectangle highlights the range of angles of the TBGs activated by the intralayer (interlayer) el–ph process. (**c**) Raman spectra of samples with angles 4.3$^{\circ }$, 5.9$^{\circ }$, 9.2$^{\circ }$ and 10.2$^{\circ }$ recorded with the excitation energies of 1.50 eV, 1.82 eV, 2.41 eV and 2.71 eV, respectively. The excitation energy for each sample corresponds to the situation of maximum resonance for the Moiré peaks, as predicted for the intralayer resonance energy represented the blue dashed line in part (**b**). (**d**) Raman spectra of TBG samples with angles 7.1$^{\circ }$, 10.2$^{\circ }$, 14.6 $^{\circ }$ and 16.3$^{\circ }$ recorded, respectively, with excitation energies 1.39 eV, 1.82 eV, 2.41 eV, and 2.66 eV. The excitation energy for each sample corresponds to the situation of maximum resonance for the Moiré peaks, as predicted for the interlayer resonance energy represented the green dashed line in part (**b**). The intensities of the TBG spectra were normalized by the intensity of the G band of single layer graphene (SLG).

We can clearly distinguish in Fig. 1a two different resonance behaviors by analysing the intensity of the G band, around 1580 ${\hbox {cm}}^{-1}$. For samples with angles between 4.3$^\circ$ and 5.9$^\circ$ the ratio of the intensities of the G bands of TBG and single layer graphene ($I_{{\text {TBG}}}/I_{{\text {SLG}}}$) is around 2, an expected result for conditions far from resonance with the van Hove singularities (vHs) in the density of states of the TBG. On the other hand, the G band is strongly enhanced for samples with angles between 7.4$^\circ$ and 10.2$^\circ$, showing in this case the occurrence of resonance with transitions between vHs in the valence and conduction bands of the TBG. The different resonance regimes shown in Fig. 1a are explained considering that the Moiré peaks in the TBG Raman spectrum can be activated by both the intralayer and interlayer resonance processes^28^, whose schematic diagrams are shown in Fig. 2a. The black and grey curves in Fig. 2a represent the Dirac cones and electronic states of the two twisted graphene layers. In the intralayer case, one incident photon with energy $E_{{\text {intra}}}$ (represented by the blue arrow) creates one electron–hole pair in one layer (in the black curve) and, then, the excited electron is scattered by a phonon with wave vector ${\mathbf{q}}_{{\text {M}}}$ (represented by the red arrow) to another state of the same layer (also in the black curve). The interlayer process is represented in the right side of Fig. 2a. Now, one incident photon with energy $E_{{\text {inter}}}$ (green arrow) creates one electron–hole pair in one layer (in the black curve) and, then, the excited electron is scattered by a phonon with wave vector ${\mathbf{q}}_{{\text {M}}}$ to a state of the other layer (in the grey curve). Notice that the interlayer resonance energy $E_{{\text {inter}}}$ coincides with the energy separation between the vHs ($E_{{\text {vHs}}}$), depicted also as a green arrow in the left side of Fig. 2a, which describes the interlayer process for phonons with ${\mathbf{q}}=0$ momentum.

The dependence of the intralayer and interlayer resonance energies ($E_{{\text {intra}}}$ and $E_{{\text {inter}}}$) on the twisting angle $\theta$ are shown as dashed blue and dashed green lines in Fig. 2b, respectively^28^. Therefore, using the dependence of $E_{{\text {intra}}}$ and $E_{{\text {inter}}}$ on $\theta$ and considering the excitation energies of each spectrum in Fig. 1a, we conclude that TBGs with angles in the range 4$^{\circ }$–6$^{\circ }$ (highlighted by the light blue strip in Fig. 1b) are in resonance with the intralayer process, whereas TBGs with angles in the range 7.5$^{\circ }$–10.2$^{\circ }$ (highlighted by the light green strip in Fig. 1b) are in resonance with the interlayer process. These conclusions are nicely in agreement with the behavior of the G band in Fig. 1a, which does not exhibit an enhancement for samples in the 4.3$^{\circ }$–5.9$^\circ$ range, a typical behavior of the intralayer resonance process, but is hugely enhanced for samples in the 7.4$^{\circ }$–10.2$^\circ$ range, as expected for the interlayer resonance. Figure 2b also shows that the Moiré peaks for the magic angle TBG ($\theta$ = 1.1$^{\circ }$) are expected to be enhanced in the Raman spectrum for excitation energies around 0.40 eV and 0.24 eV for the intralayer and interlayer processes, respectively, which are much smaller that the excitation energies used in this work.

Let us now discuss how the intralayer and interlayer processes enhance the distinct phonons of graphene. We can observe in Fig. 1a,b that only phonons of the TA and the LO branches appear in the case of the intralayer resonance, whereas phonons from all branches (ZA, TA, LA, oTO, iTO and LO) are enhanced by the interlayer resonance process. Figure 2c shows the Raman spectra of four different samples of TBGs with angles 4.3$^{\circ }$, 5.9$^{\circ }$, 9.2$^{\circ }$ and 10.2$^{\circ }$ in the region of the iTO and LO peaks recorded with the excitation energies of 1.50 eV, 1.82 eV, 2.41 eV and 2.71 eV, respectively. The excitation energy for each sample corresponds to the situation of maximum resonance observed for the Moiré peaks. Notice that the ratio of the intensities of the G band in TBG and single layer graphene ($I_{{\text {TBG}}}/I_{{\text {SLG}}}$) in Fig. 2c is always around two, showing thus that the Moiré peaks are enhanced by the intralayer el–ph process. Interestingly, the intensity of the LO peak at resonance condition strongly depends on the twisting angle $\theta$. For example, in the case of the 4.3$^{\circ }$ sample in Fig. 2c, it becomes four times more intense than the G band. For the $\theta$ = 5.9$^{\circ }$ sample, the LO peak is not so strong but still more intense than the G band. For the 9.2$^{\circ }$ sample the LO peak is much weaker that the G band and, in the case of the 10.2$^{\circ }$ sample, we can only observe a weak feature at the position of the LO peak in Fig. 2c. Concerning the activation of the iTO phonons by the intralayer process, we do not observe any peak associated with the iTO phonons in the spectra of TBG samples in resonance with the intralayer process, as illustrated for the cases shown in Fig. 2c. Our results show that intralayer el–ph process does not enhance the iTO phonons in the Raman spectra of TBG samples with small and intermediate twisting angles, in agreement with previously reported Raman results in TBGs^22,28,30^

Figure 2d shows the Raman spectra of four different samples of TBGs with angles 7.1$^{\circ }$, 10.2$^{\circ }$, 14.6$^{\circ }$ and 16.3$^{\circ }$ in the region of the iTO and LO peaks recorded, respectively, with the excitation energies of 1.39 eV, 1.82 eV, 2.41 eV, and 2.66 eV. The excitation energy for each sample corresponds to the situation of maximum resonance observed for the Moiré peaks. We can now observe a huge enhancement of the G band of the TBGs, which becomes at resonance condition up to 80 times more intense than the G band of single layer graphene, as shown by the dashed curves in Fig. 2d. In the case of the 7.1$^{\circ }$ and 10.2$^{\circ }$ samples, we can clearly observe in Fig. 2d the iTO and LO peaks below and above the G band, respectively, showing that the interlayer el–ph process enhances both the iTO and LO phonons of graphene. However, the enhancement of the LO peak is weaker in the interlayer case when compared with the intralayer el–ph process shown in Fig. 2c. Notice that the LO peak cannot be observed in the spectra of the 14.6$^{\circ }$ and 16.3$^{\circ }$ samples, as shown in Fig. 2d. The LO frequency is closer to the G band for these angles (see Fig. 1b) and the appearance of the LO peak in the spectra may be masked by the huge enhancement of the G band.

### The double-resonance Raman model for the intralayer and interlayer el–ph processes

In the last sections, we have shown that the intralayer and interlayer processes enhance phonons from different branches in a distinct way and that some phonon branches are not enhanced by the intralayer resonance process. Now we will address these points considering the double-resonance (DR) Raman model for graphene and the specific momentum conservation conditions for the intralayer and interlayer electron–phonon processes. In graphene, phonons with ${\mathbf{q}}\ne 0$ can be accessed through the DR Raman process either by a defect induced mechanism or by a scattering of two phonons with opposite momenta ${\mathbf{q}}$ and $-{\mathbf{q}}$^7,16^. The intralayer process in TBG can be considered as a DR mechanism that occurs in one graphene layer where now the momentum conservation is guaranteed by the Moiré vector ${\mathbf {q}}_{{\text {M}}}(\theta )$ instead of being provided by a defect or an additional phonon. We can make a parallel between the LO intralayer process in TBG with the defect-induced ${\hbox {D}}^{\prime }$ band in graphene, around 1620 ${\hbox {cm}}^{-1}$^16^. The ${\hbox {D}}^{\prime }$ band originates from the intravalley DR Raman process in graphene involving one phonon of the LO branch, where momentum conservation for the process is provided by a defect. For a TBG, momentum conservation is provided by the periodic potential generated in one layer by the other one twisted by $\theta$.

It is worth to mention that the iTO phonons are not activated by the intravalley double resonance process in disordered graphene, and this result was ascribed to the destructive quantum interference effect that suppress their contribution in the spectrum^27^. Additionally, it is known that the wave vectors in the BZ of graphene along the $\Gamma -K-M$ direction belong to the ${\hbox {C}}_{{2v}}$ point group and their symmetries are labeled by the letter *T*. The electronic bands have either $T_2$ or $T_4$ symmetries^31,32^. Two excited states in the same valley have necessarily different symmetries. Pair of excited states with same symmetry ($T_2$ or $T_4$) occurs only if electrons are in different valleys, around the *K* and $K^{\prime }$ points. Along this high-symmetry direction, the iTO and LA phonons have $T_1$ symmetry and the LO and TA phonons have $T_3$ symmetry^31,32^. Group theory shows that electronic transitions between excited states of same symmetry require a $T_1$-symmetry phonon, whereas a $T_3$-symmetry phonon only allows transitions between excited states of different symmetries. Therefore, the intravalley DR process along the $\Gamma -K-M$ direction, where the excited states have different symmetries, involves only LO and TA phonons, that have $T_3$ symmetry. The experimental results in this work for the intralayer resonance enhancement in small angle TBGs are in agreement with the group-theory predictions along the high symmetry $\Gamma -K-M$ direction. However, the lack of observation of the iTO peaks even in small twisting angles regime cannot be explained solely by group-theory, since the direction of the ${\mathbf{q}}_{{\text {M}}}$ mapped on the BZ of one graphene layer always deviate from the high-symmetry $\Gamma -K$ line for $\theta \ne 0$ (dotted white lines in panels of the Fig. 4).

**Figure 3:**
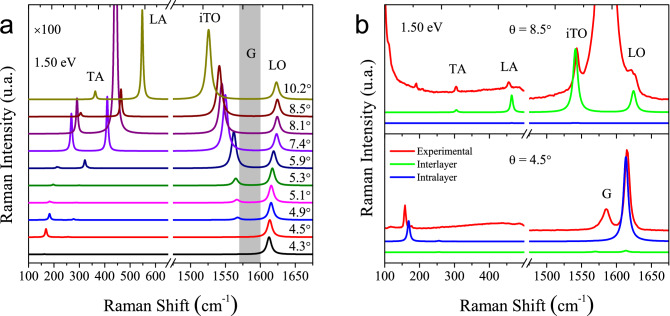
(**a**) Theoretical total Raman intensity for TBGs with the same angles of the experimental samples, between 4.3$^\circ$ and 10.2$^\circ$. All spectra were calculated using the double-resonant Raman model described in the text, which includes interlayer and intralayer el–ph processes, for the excitation energy of 1.50 eV. The intensities in the acoustic region ($\omega <700$ ${\hbox {cm}}^{-1}$), which shows the peaks associated to TA and LA modes, were enhanced by a factor of 100 for a better comparison with the optical region ($\omega >1400$ ${\hbox {cm}}^{-1}$), that shows the iTO and LO peaks. The grey strip between 1570 ${\hbox {cm}}^{-1}$ and 1600 ${\hbox {cm}}^{-1}$ denotes the region of the G band, which is a first-order process not included in the calculations. (**b**) Theoretical Raman spectra of TBG structures for $\theta = 8.5^\circ$ and 4.5$^\circ$ showing separately the intralayer (blue curves) and interlayer (green curves) processes, for the excitation energy of 1.50 eV, compared with experimental measurements (red curves). In this case, the acoustic region was magnified by a factor of 50 compared to the optical one and all the theoretical frequencies shown in the figures were shifted by $\pm 20$ ${\hbox {cm}}^{-1}$, in the optical and the acoustic region, respectively.

In the DR model, the Raman intensity related to a phonon belonging to branch $\nu$ in the phonon dispersion is calculated by means of fourth-order time-dependent perturbation theory. Here we consider a sum over all electron wave vectors (${\mathbf {k}}$) in the SLG BZ, but we take in account only phonons with wave vectors equal to ${\mathbf{q}}_{{\text {M}}}$, for a given twisting angle.

1 $$\begin{aligned} I_{\nu }({\mathbf {q}}_{{\text {M}}}) \propto \left |\sum _{{\mathbf {k}}} \frac{{\mathscr {M}}_{Cf} {\mathscr {M}}_{BC} {\mathscr {M}}_{AB} {\mathscr {M}}_{iA}}{[\varepsilon _L-\varepsilon _A -i\gamma ] [\varepsilon _L-\varepsilon _B -i\gamma ] [\varepsilon _L-\varepsilon _C -i\gamma ] }\right |^2. \end{aligned}$$

The expression above corresponds to the Fermi golden rule transition probability for the 4-th order process^27,33^, where the system in an initial state *i* goes through three intermediate states *A*, *B* and *C*, to a final state *f*. The terms in the numerator corresponds to the matrix elements of the four processes: from right to left, ${\mathscr {M}}_{iA}$ corresponds to the matrix element of the absorption of light and creation of an electron–hole pair, ${\mathscr {M}}_{AB}$ corresponds to the matrix element of the electron–phonon (el–ph) interaction that scatters the carriers from *A* to *B*, ${\mathscr {M}}_{BC}$ is related elastic scattering provided by the Moiré potential, and ${\mathscr {M}}_{Cf}$ corresponds to the matrix element of the recombination of the electron–hole pair and emission of the scattered photon. $\varepsilon _L$ is the excitation energy, $\varepsilon _A$, $\varepsilon _B$ and $\varepsilon _C$ are the energies of states *A*, *B* and *C*, respectively, and $\gamma$ is the broadening energy related to the finite lifetime of the intermediate states. In this work ${\mathscr {M}}_{iA}$, ${\mathscr {M}}_{Cf}$, $\varepsilon _A$, $\varepsilon _B$, $\varepsilon _C$ and $\gamma$ are calculated exactly as in Ref.^27^ and ${\mathscr {M}}_{BC}$ is considered as a constant.

The el–ph matrix element is given by ${\mathscr {M}}_{AB} = \langle {\mathbf {k}}^{\prime } |\Delta H_{{\mathbf {q}},\nu }|{\mathbf {k}}\rangle$ and it connects the electronic states $|{\mathbf {k}}\rangle$ and $|{\mathbf {k}}^{\prime }\rangle$. $\Delta H_{{\mathbf {q}},\nu }$ is the derivative of the Hamiltonian with respect to the periodic displacement related to the phonon from branch $\nu$ with wave vector ${\mathbf {q}}$^27^. In the intralayer process, the electronic state $|{\mathbf {k}}\rangle$ in one layer differs from the state $|{\mathbf {k}}^{\prime }\rangle$ in the same layer by the Moiré vector ${\mathbf {q}}_{{\text {M}}}$, as depicted in Fig. 2a, while in the interlayer process the difference is given by a rotation of $\theta$, since $|{\mathbf {k}}\rangle$ and $|{\mathbf {k}}^{\prime }\rangle$ are in different layers, followed by a scattering with a Moiré vector ${\mathbf {q}}_{{\text {M}}}$. In other words, we consider the conditions:

2 $$\begin{aligned} {\mathbf {k}}^{\prime }_{{\text {intra}}} ={\mathbf {k}}+{\mathbf {q}}_{{\text {M}}}(\theta ) \end{aligned}$$

and

3 $$\begin{aligned} {\mathbf {k}}^{\prime }_{{\text {inter}}}=R(\theta ){\mathbf {k}} + {\mathbf {q}}_{{\text {M}}}(\theta ) \end{aligned}$$

for the intralayer and interlayer processes, respectively, being $R(\theta )$ the rotation matrix.

Figure 3a shows the total calculated Raman intensity obtained by the sum of intralayer and interlayer processes described above, for $\theta$ between 4.3$^\circ$ and 10.2$^\circ$. We show here only the results for excitation energy of 1.50 eV, but the main results are qualitatively similar for excitation energies in the NIR and visible range. The overall agreement between calculations of Fig. 3a and the experimental results shown in Fig. 1a is clear. In particular, we can also distinguish in the calculations the two regimes where the intralayer or interlayer el–ph mechanisms are more relevant. For angles between 4.3$^\circ$ and 5.3$^\circ$ we see that the peaks associated with the TA and LO modes are more intense than the LA and iTO ones, respectively, in agreement with the experimental results. On the other hand, for angles between 5.9$^\circ$ and 10.2$^\circ$, the TA, LA, iTO and LO modes have all non negligible intensities. All spectra were normalized by the respective LO intensity, but the acoustic (TA and LA) peaks were multiplied by a factor of 100 for a better visualisation. Since we do not consider out-of-plane polarized light, the ZA and oTO modes cannot be described in this model. We remark that the G band is a single-resonant process, which is not included in the present calculations (the vertical grey strip in Fig. 3a represents the region where the G band is supposed to appear in the spectra).

In Fig. 3b we plot separately the calculated spectra considering the contribution of the interlayer (green curves) and intralayer (blue curves) processes for TBGs with twisting angles of 4.5$^\circ$ (bottom panel) and 8.5$^\circ$ (upper panel), and the experimental spectra (red curves) for the same angles shown in Fig. 1a. Notice that the enhancement of the peaks and the relative intensities between the longitudinal and transverse components of the acoustic and optical phonons follow the same trend observed in the experimental Raman spectra. For the 4.5$^\circ$ sample, we only observe the enhancement of the TA and LO peaks by the intralayer process whereas, for the 8.5$^\circ$ sample, the enhancement is provided by the interlayer el–ph process and phonons of the TA, LA, iTO and LO branches are activated. These results show that, indeed, the intralayer process is stronger for small angle TBGs, while the enhancement for TBGs with intermediate angles is dominated by the interlayer el–ph process.

**Figure 4:**
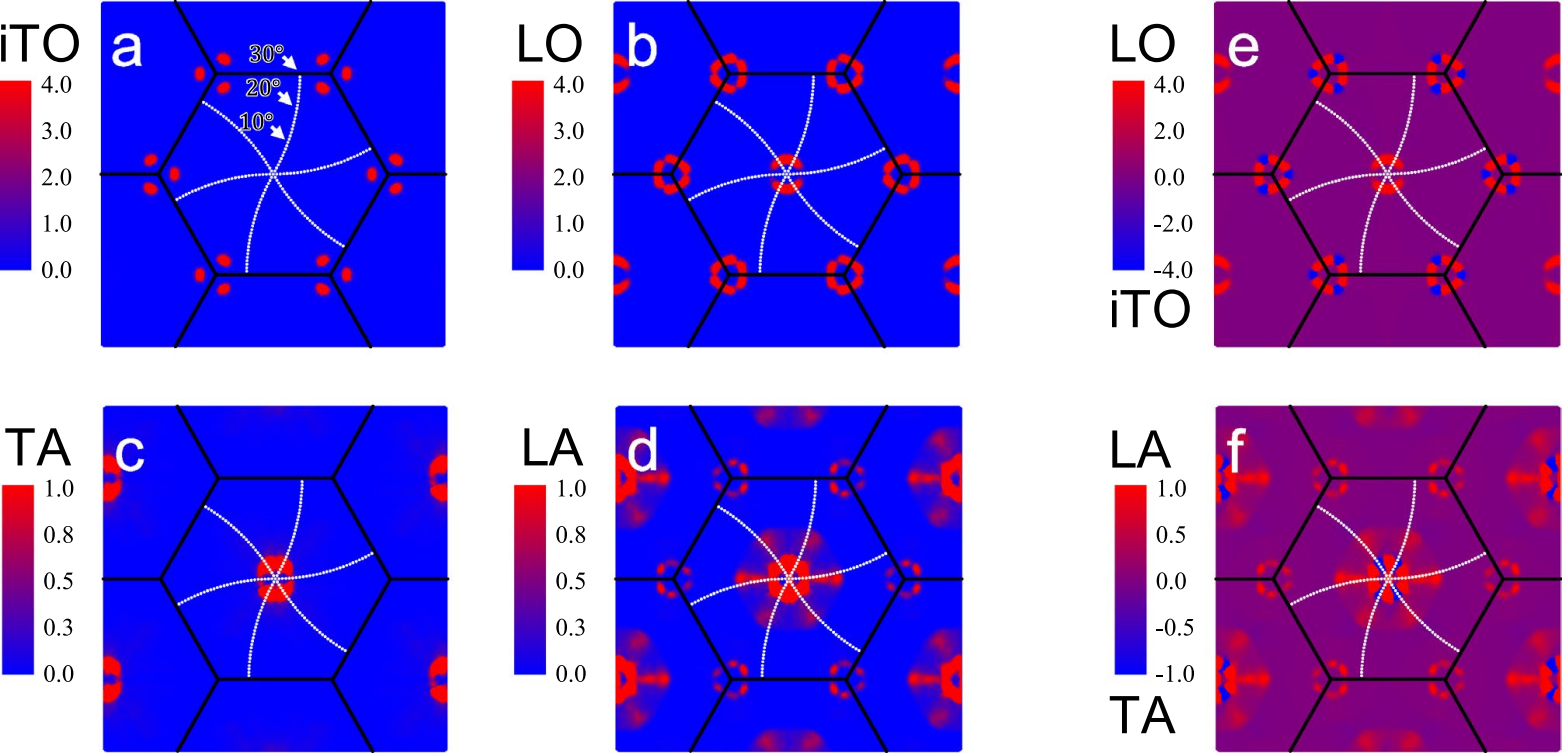
(**a**–**d**) Theoretical double-resonance Raman intensity ${\tilde{I}}_{\nu }({\mathbf {q}})$ in graphene as a function of the phonon wave vector ${\mathbf {q}}$ for the iTO, LO, TA and LA branches, calculated without the restriction ${\mathbf {q}}$ = ${\mathbf {q}}_{{\text {M}}}$. The white dotted lines in these figures represent the Moiré vector ${\mathbf {q}}_{{\text {M}}}$ paths as function of the twisting angle, being $\theta =0$ at the $\Gamma$ point and $\theta =30^{\circ }$ at the middle point between *K* and *M*. (**e**–**f**) Differences between ${\tilde{I}}_{\nu }({\mathbf {q}})$ of longitudinal and transverse branches, ${\tilde{I}}_{{\text {LO}}}({\mathbf {q}})-{\tilde{I}}_{{\text {iTO}}}({\mathbf {q}})$ and ${\tilde{I}}_{{\text {LA}}}({\mathbf {q}})-{\tilde{I}}_{{\text {TA}}}({\mathbf {q}})$.

In order to achieve a deeper understanding regarding the contribution of each phonon branch for the calculations shown in Fig. 3a,b, we have also performed calculations using Eq. (1) considering all phonon wave vectors ${\mathbf {q}}$, without restricting the calculation to the Moiré wave vectors (${\mathbf {q}}_{{\text {M}}}$). The intensities of the DR process ${\tilde{I}}_{\nu }({\mathbf {q}})$ as a function of the phonon wave vector ${\mathbf {q}}$, where $\nu$ represents the iTO, LO, TA and LA phonon branches, are shown in Fig. 4a–d for all phonon wave vectors ${\mathbf {q}}$ within the graphene BZ represented by the black hexagons. The white dotted lines in these figures represent the Moiré vector ${\mathbf {q}}_{{\text {M}}}$ paths as function of the twisting angle, being $\theta =0$ at the $\Gamma$ point and $\theta =30^{\circ }$ at the middle point between *K* and *M*. We see in Fig. 4a,b that the iTO peak intensity for **q**-vectors close to the $\Gamma$ point is almost null, while the LO peak is very intense in this region. For the acoustic peaks shown in Fig. 4c,d, we see that the TA and LA peaks are more intense close to $\Gamma$, indicating that both peaks could be seen in the Raman spectra for small $\theta$ TBGs. Furthermore, we have computed the differences between ${\tilde{I}}_{\nu }({\mathbf {q}})$ of longitudinal and transverse branches. Figure 4e shows this difference for LO and iTO branches, ${\tilde{I}}_{{\text {LO}}}({\mathbf {q}})-{\tilde{I}}_{{\text {iTO}}}({\mathbf {q}})$, and Fig. 4f shows ${\tilde{I}}_{{\text {LA}}}({\mathbf {q}})-{\tilde{I}}_{{\text {TA}}}({\mathbf {q}})$. We can observe in Fig. 4e,f that there is a transition between the most intense peak as long as the **q**-vectors follow the Moiré path. The blue regions present a higher intensities of the iTO (TA) peak with respect to the LO (LA), while the red regions show the opposite. The intermediate situation, where the intensities of the iTO and LO (TA and LA) peaks are comparable to each other, is represented by the purple color. Figure 4e,f show that TA and LO are the most intense peaks in the spectra when $\theta$ is small (DR processes close to $\Gamma$) but, for intermediate twisting angles, both longitudinal and transverse components of the acoustic and optical peaks have comparable intensities. We can thus conclude that the experimental results can be very well explained and understood by the double resonance (DR) Raman calculations for graphene considering momentum conservation provided by the Moiré potential and the intralayer and interlayer el–ph resonance conditions.

## Conclusion

The Raman spectra of twisted bilayer graphene exhibit a number of extra peaks associated with phonons of the acoustic and optical branches of graphene with the wave vector of the Moiré pattern, which can be activated by both the intralayer and the interlayer electron–phonon processes. In this work, we studied how the intralayer and the interlayer el–ph processes enhance the intensity of each extra peak in the Raman spectrum of twisted bilayer graphene (TBG). CVD grown samples of TBGs with different twisting angles $\theta$ in the range 4$^\circ$–16$^\circ$ were measured using several laser excitation energies in the range 1.39–2.71 eV. As predicted by the dependence of the intralayer and interlayer resonance energies on the twisting angle $\theta$, resonance Raman behavior were observed in different samples with angles in the range 4.3$^\circ$–10.2$^\circ$ for the intralayer el–ph process and in the range 7.4$^\circ$–16.3$^\circ$ for the interlayer el–ph process. Usually twisting angles are estimated by the optical images of the samples, leading to uncertainties of the order of 1$^\circ$. Here, we present a more accurate procedure where the twisting angle is obtained from the positions of the TA and LA peaks. The angle $\theta$ is determined from the simultaneous fit of the positions $f_{{\text {TA}}}$ and $f_{{\text {LA}}}$ of the TA and LA peaks, respectively, by the expressions $f_{{\text {TA}}}$ = 4040 $\sin (\theta /2)$ ${\hbox {cm}}^{-1}$ and $f_{{\text {LA}}}$ = 6232 $\sin (\theta /2)$ ${\hbox {cm}}^{-1}$.

Peaks associated with all phonon branches of graphene were observed experimentally in the Raman spectra of TBG samples in resonance with the interlayer process. They can be as intense as the G band of single layer graphene, but much weaker than the TBG G band. Their positions are nicely described by the calculated dependence of phonon frequencies on $\theta$. On the other hand, our experimental results shows that the intralayer process enhances preferentially phonons of the TA and LO branches, considering the laser energies and the range of $\theta$ of the samples used in this work. The Raman enhancement by the intralayer process is very intense and the Moiré peak can be much stronger than the G band at resonance condition. It was observed that the enhancement of the LO peak increases with decreasing twisting angle $\theta$. The experimental results are explained by theoretical calculations of the double-resonance Raman process in graphene, within the fourth-order perturbation theory approach, considering the momentum conservation provided by the Moiré vector ${\mathbf {q}}_{{\text {M}}}(\theta )$ for both the intralayer and interlayer el–ph processes. The observed enhancement of the acoustic and optical phonons by the intralayer and interlayer processes was nicely reproduced in the calculated DR spectra, showing the importance of taking into account the symmetry-related selection rules for the electron–phonon interaction in graphene, as well as the quantum interference that affect the intensities of the Raman peaks activated by the Moiré superlattice.

## Methods

Raman measurements of the TBG samples with different twisting angles $\theta$ were performed using several laser lines in the near infrared (NIR) and visible ranges. Experiments in the visible range were performed in a DILOR XY triple-monochromator spectrometer equipped with a N2-cooled charge-couple device detectors, using an Ar/Kr laser with 12 excitation energies in the visible range. The NIR Raman measurements were performed on a home-made setup, including an iHR-550 Horiba spectrometer equipped with a liquid-nitrogen-cooled silicon CCD detector. A tunable CW Ti:sapphire laser filtered using tunable laser line filters^34^ was used for excitation. The scattered light was collected through a $\times \, 100$ objective (NA = 0.95) using a backscattering configuration.

The TBG samples were grown by CVD technique using methane (99.99 per cent) on polycrystalline Cu foils^30^. The graphene layers were first covered by a thin layer of polycarbonate, followed by etching in HCl aqueous solution to remove the Cu in the transfer process. The polycarbonate film with attached graphene was then transferred onto fused silica. Finally, the polycarbonate film was removed using chloroform.

The double-resonance (DR) Raman intensities were computed in the fourth order perturbation theory. The eigenenergies and the eigenstates were obtained through a fifth neighbour tight-binding model fitted to a DFT + GW calculations, as well as the phonon dispersion used in all calculations. The electronic wave vectors were distributed in a $480 \times 480$ **k**-point grid over a graphene BZ and only phonon wave vectors ${\mathbf {q}}$ which conserve momentum with the Moiré vector ${\mathbf {q}}_{{\text {M}}}(\theta )$ for a given twisting angle $\theta$ were used in TBG, while in the full DR Raman calculation in SLG, a $240 \times 240$ **q**-point grid for all six phonon branches were used. Despite the restrictions associated to the interlayer and intralayer processes, all Raman calculations were performed following the procedures described in Ref.^27^.
